# SARS-CoV-2 Incubation Period during Omicron BA.5–Dominant Period, Japan (Response)

**DOI:** 10.3201/eid3001.231487

**Published:** 2024-01

**Authors:** Tsuyoshi Ogata, Hideo Tanaka

**Affiliations:** Itako Public Health Center of Ibaraki Prefectural Government, Ibaraki, Japan (T. Ogata);; Public Health Center of Neyagawa City, Osaka, Japan (H. Tanaka)

**Keywords:** COVID-19, respiratory infections, severe acute respiratory syndrome coronavirus 2, SARS-CoV-2, SARS, incubation period, Omicron, BA.5, coronavirus disease, zoonoses, viruses, coronavirus, Japan

**In Response:** We thank Dr. Cheng and colleagues (*1*) for their valuable comments regarding our study of incubation periods observed for the SARS-CoV-2 Omicron BA.5 subvariant in Japan (*2*). As we indicated in our study limitations paragraph, “patient pairs with long incubation periods might be censored during observational periods, and selection bias might result in underestimation” (*2*). We have several other comments to make regarding our study. First, our previous study during the increasing dominance of the Omicron BA.1 subvariant only included patients who had 1 exposure day; we reported incubation periods of 3.0 days for L452R mutation–negative patients and 3.3 days for unvaccinated patients (*3*), which was similar to 3.2 days reported in a study of patients with BA.1 infections who had multiple exposure days (*4*). Therefore, the effect of only including patients who had 1 exposure day should be further evaluated. Second, the incubation period for the BA.5 subvariant in our study was 3.0 days for patients with infectors who were ≤19 years of age and 2.1 days for patients with infectors who were ≥60 years of age (*2*). Because those data are considerably different, adjustment for demographic factors for both infectors and infectees might be necessary to compare incubation periods. Third, although including patients with multiple exposure days decreases selection bias, it might increase uncertainty regarding the actual time of infection (*5*). Therefore, comparing incubation periods in studies that use various methods and evaluating corresponding study limitations are useful for review and discussion.
